# Selective histone deacetylase inhibition as a therapeutically relevant strategy in H3K27M-glioma

**DOI:** 10.1093/neuonc/noag142

**Published:** 2026-06-25

**Authors:** Andrew Groves, Orville O Kirkland, Costanza Lo Cascio, Xiuqi Wang, Caleb Heaslip, Joana Graca Marques, Chen Song, Rebecca Poetschke, Tingjian Wang, Diana H Chin, Yaw Asante, Qi Liu, Hyunmin Kim, Shanqing Gu, Maria Trissal, Jenna Labelle, Gustavo Alencastro Veiga Cruzeiro, Kristen L Jones, Amy B Cameron, Louise M Clark, Brittaney Leeper, Quang-De Nguyen, Berkley E Gryder, Mariella G Filbin, Jun Qi

**Affiliations:** Dana-Farber/Boston Children’s Cancer and Blood Disorders Center, Harvard Medical School, Boston, Massachusetts, USA; Broad Institute of Harvard and MIT, Cambridge, Massachusetts, USA; Division of Hematology/Oncology, University of Iowa Stead Family Children’s Hospital, Iowa City, Iowa, USA; Department of Cancer Biology, Dana-Farber Cancer Institute, Boston, Massachusetts, USA; Department of Medicine, Harvard Medical School, Boston, Massachusetts, USA; Department of Chemistry & Chemical Biology, Harvard University, Cambridge, Massachusetts, USA; Dana-Farber/Boston Children’s Cancer and Blood Disorders Center, Harvard Medical School, Boston, Massachusetts, USA; Broad Institute of Harvard and MIT, Cambridge, Massachusetts, USA; Department of Cancer Biology, Dana-Farber Cancer Institute, Boston, Massachusetts, USA; Department of Medicine, Harvard Medical School, Boston, Massachusetts, USA; Department of Cancer Biology, Dana-Farber Cancer Institute, Boston, Massachusetts, USA; Department of Medicine, Harvard Medical School, Boston, Massachusetts, USA; Dana-Farber/Boston Children’s Cancer and Blood Disorders Center, Harvard Medical School, Boston, Massachusetts, USA; Broad Institute of Harvard and MIT, Cambridge, Massachusetts, USA; Department of Cancer Biology, Dana-Farber Cancer Institute, Boston, Massachusetts, USA; Department of Medicine, Harvard Medical School, Boston, Massachusetts, USA; Dana-Farber/Boston Children’s Cancer and Blood Disorders Center, Harvard Medical School, Boston, Massachusetts, USA; Broad Institute of Harvard and MIT, Cambridge, Massachusetts, USA; Department of Cancer Biology, Dana-Farber Cancer Institute, Boston, Massachusetts, USA; Department of Medicine, Harvard Medical School, Boston, Massachusetts, USA; Department of Genetics and Genome Sciences, Case Western Reserve University School of Medicine, Cleveland, Ohio, USA; Department of Nutrition, Case Western Reserve University School of Medicine, Cleveland, Ohio, USA; Department of Cancer Biology, Dana-Farber Cancer Institute, Boston, Massachusetts, USA; Department of Medicine, Harvard Medical School, Boston, Massachusetts, USA; Department of Genetics and Genome Sciences, Case Western Reserve University School of Medicine, Cleveland, Ohio, USA; Department of Genetics and Genome Sciences, Case Western Reserve University School of Medicine, Cleveland, Ohio, USA; Dana-Farber/Boston Children’s Cancer and Blood Disorders Center, Harvard Medical School, Boston, Massachusetts, USA; Broad Institute of Harvard and MIT, Cambridge, Massachusetts, USA; Dana-Farber/Boston Children’s Cancer and Blood Disorders Center, Harvard Medical School, Boston, Massachusetts, USA; Broad Institute of Harvard and MIT, Cambridge, Massachusetts, USA; Dana-Farber/Boston Children’s Cancer and Blood Disorders Center, Harvard Medical School, Boston, Massachusetts, USA; Broad Institute of Harvard and MIT, Cambridge, Massachusetts, USA; Lurie Family Imaging Center, Center for Biomedical Imaging in Oncology, Dana-Farber Cancer Institute, Boston, Massachusetts, USA; Lurie Family Imaging Center, Center for Biomedical Imaging in Oncology, Dana-Farber Cancer Institute, Boston, Massachusetts, USA; Lurie Family Imaging Center, Center for Biomedical Imaging in Oncology, Dana-Farber Cancer Institute, Boston, Massachusetts, USA; Lurie Family Imaging Center, Center for Biomedical Imaging in Oncology, Dana-Farber Cancer Institute, Boston, Massachusetts, USA; Lurie Family Imaging Center, Center for Biomedical Imaging in Oncology, Dana-Farber Cancer Institute, Boston, Massachusetts, USA; Department of Genetics and Genome Sciences, Case Western Reserve University School of Medicine, Cleveland, Ohio, USA; Dana-Farber/Boston Children’s Cancer and Blood Disorders Center, Harvard Medical School, Boston, Massachusetts, USA; Broad Institute of Harvard and MIT, Cambridge, Massachusetts, USA; Department of Cancer Biology, Dana-Farber Cancer Institute, Boston, Massachusetts, USA; Department of Medicine, Harvard Medical School, Boston, Massachusetts, USA

**Keywords:** diffuse intrinsic pontine glioma | diffuse midline glioma | epigenetics, histone deacetylase inhibitor, pediatric brain tumor

## Abstract

**Background:**

Diffuse midline glioma (DMG) is a lethal pediatric brain tumor, driven by profound epigenetic dysregulation, raising the possibility that targeting epigenetic regulators such as histone deacetylases (HDACs) could provide a therapeutic benefit. However, despite preclinical activity, currently available HDAC inhibitors have shown limited clinical translation due to poor central nervous system (CNS) penetration and a lack of HDAC isoform selectivity.

**Methods:**

We combined CRISPR/Cas9 screening and pharmacologic HDAC inhibition to identify isoform-specific dependencies in DMG cell lines. Mechanistic studies were performed using bulk RNA-seq and HiChIP, and therapeutic efficacy was evaluated in orthotopic patient-derived xenograft mouse models.

**Results:**

We identify that the nuclear-localized Class I HDAC isoforms are dependencies in DMG and demonstrate that HDAC2 represents a selective dependency that is highly expressed in patient tumors. We further identify a CNS-penetrant HDAC inhibitor, Compound 26, with preferential HDAC1/2 isoform selectivity. Transcriptomic and chromatin conformation analyses reveal that Compound 26 induces rapid transcriptional reprogramming, restores physiologic pontine histone patterns, and induces cell cycle arrest through chromatin reorganization. Consistent with these effects, Compound 26 slows DMG growth *in vitro* and in orthotopic *in vivo* models.

**Conclusions:**

Our findings establish HDAC2 as a critical and therapeutically actionable dependency in DMG. Genetic or pharmacological targeting of HDAC2 disrupts DMG chromatin architecture and induces cell cycle arrest. The HDAC1/2-selective inhibitor Compound 26 exhibits favorable CNS-penetration, tolerability, and antitumor activity in disease-relevant patient-derived xenograft models. Collectively, these data support isoform-selective HDAC inhibition as a rational strategy to overcome the translational limitations of pan-HDAC inhibitors in DMG.

Key Points

- HDAC class I isoforms are genetic and chemical dependencies in DMG.
- Selective inhibition of these isoforms exploits epigenetic vulnerabilities to trigger cell cycle arrest.
- The CNS penetrant, Class I selective HDAC inhibitor Compound 26 is effective *in-vivo*.

Importance of the Study

Epigenetic reprogramming via histone deacetylase (HDAC) inhibition has shown promise in models of diffuse midline glioma (DMG); however, clinical outcomes with HDAC inhibitors (HDACi) have thus far been disappointing. These limitations reflect multiple factors, including poor isoform selectivity, inadequate central nervous system (CNS) penetration of agents in clinical development, and an incomplete mechanistic understanding of HDAC biology in DMG. Here, we identify Class I HDAC isoforms as critical dependencies in DMG, with HDAC2 emerging as a selective vulnerability. We demonstrate that selective inhibition of these isoforms with a CNS-penetrant HDAC inhibitor, Compound 26, disrupts DMG chromatin architecture and induces cell-cycle arrest. Importantly, Compound 26 is well-tolerated *in vivo*, reduces tumor proliferation, and extends survival in orthotopic patient-derived xenograft models. Collectively, this study provides a mechanistic and translational framework for isoform-selective HDAC targeting to overcome the limitations of pan-HDAC inhibitors in DMG.

Diffuse midline glioma (DMG) is a lethal childhood brain tumor, with limited effective treatment options and a dismal median overall survival of approximately 11 months.^1^ The majority of DMGs harbor a unique heterozygous lysine-to-methionine (K27M) mutation in histone H3.3 or H3.1, leading to widespread epigenetic dysregulation.^2^^,^^3^ Downstream hallmark consequences of this mutation include both global reduction in the repressive H3K27me3 mark, caused by inhibition of the main H3K27 methyltransferase, enhancer of zeste homolog 2 (EZH2), and dysregulated histone acetylation.^4^^,^^5^

Histone acetylation is tightly regulated by the opposing actions of histone acetyltransferases (HATs) and histone deacetylases (HDACs). Eighteen HDAC isoforms have been identified in human cells and grouped into 4 classes (I, II, III, and IV) according to their sequence homology, subcellular localization, co-factor requirement, and evolutionary relationship to yeast enzymes.^6^ Among these, HDAC1/2/3 are the most ubiquitously expressed and account for most histone deacetylation activity within chromatin. The remaining HDAC family members primarily target non-histone substrates, and their activity toward histones tend to be tissue-specific and context-dependent.

HDAC1 and HDAC2, both Class I HDACs, share nearly identical catalytic core domains and frequently operate together in transcriptional repressor complexes such as CoREST, NuRD, and Sin3A.^7^ These shared complexes underpin many of their overlapping functions in regulating gene expression programs critical for cell cycle progression, differentiation, and oncogenesis.^8^ However, despite their high structural similarity, HDAC1 and HDAC2 also execute distinct, non-redundant and highly context-specific functions, particularly in the brain. For example, HDAC2 alone is both essential and sufficient to support normal brain development and survival in the absence of HDAC1, as demonstrated in Nestin-Cre-driven conditional knockout mice.^9^ Consistent with this developmental specialization, several studies indicate that HDAC1 and HDAC2 likewise perform non-overlapping functions in glioblastoma (GBM).^10^^,^^11^ However, whether such specialized, context-specific functions are preserved in DMG remains unknown.

In general, the majority of FDA-approved HDACi have broad-spectrum activity inhibiting all HDAC isoforms (“pan-HDAC”), and as such have potent effects on normal tissues, leading to systemic toxicity and a narrow therapeutic window.^12^ An additional problem for CNS tumors is the challenge of drug delivery across the blood-brain barrier (BBB), which likely contributed to the failure of several HDACi to demonstrate meaningful benefit in primary brain tumors.^13^ For example, the FDA-approved pan-HDACi panobinostat is effective in patient-derived DMG cell lines as well as orthotopic animal models.^14^ However, a phase I trial in children with DMG (PBTC-047) was terminated due to dose-limiting myelosuppression from systemic pan-HDAC inhibition, without any observed clinical benefit, likely reflecting panobinostat’s poor CNS penetration.^15-17^ These findings highlight a deeper conceptual problem, which is that the functional importance of individual HDAC isoforms in DMG remains largely unknown, including which isoforms are most highly expressed and which are required for tumor maintenance. In the absence of this knowledge, the rationale for using broad-spectrum pan-HDAC inhibition remains insufficiently defined, particularly given the toxicity-driven constraints that prevent achieving therapeutic CNS exposure.

Here, we address these challenges by performing comprehensive chemogenomic profiling to assess the functional importance of individual HDAC isoforms in several DMG cell lines. We discover that HDAC2 is an essential dependency for DMG proliferation and identify a Class I-selective and CNS-penetrant HDACi, termed “Compound 26,” that potently inhibits the growth of several patient-derived DMG cell lines. Compound 26 treatment of DMG cell lines causes hyperacetylation and chromatin structural rewiring, resulting in G1 cell cycle arrest. CNS pharmacokinetic (PK) and pharmacodynamic (PD) profiling confirms that Compound 26 effectively crosses the BBB. Importantly, Compound 26 is well tolerated and significantly prolongs survival in an aggressive DMG orthotopic mouse model.

## Methods

### Cell Culture

Patient derived H3K27M-glioma cell lines established at the Boston Children’s Hospital, Stanford University and the Hospital Sant Joan de Deu Barcelona were maintained as previously described.^18^

### Drug Testing and Cell Viability Assays

For viability testing, single cells (800 cells per well) were plated in a 384-well untreated, white-wall, clear-bottom plates. Cells were dosed the next day and at 96 hours post-plating with compounds using a D300e digital dispenser (HP). CellTiter-Glo assay was performed as per the manufacturer’s instructions (Promega) using an Envision plate reader (PerkinElmer, USA).

### HDAC Isoform Inhibition Assay

Fluorogenic HDAC isoform inhibition assay for all indicated HDAC inhibitors was conducted as previously described.^19^ Recombinant HDAC protein was obtained from BPS Bioscience.

### Apoptosis and Cell Cycle Assays

Flow cytometry was performed on a BD LSR-II flow cytometer. Apoptosis was assessed using annexin V and 7AAD staining according to the manufacturer’s instructions (Biologened, Cat # 640924). Proliferation and cell cycle assessment was performed using the Click-iT EdU kit (Thermo Fisher) with FxCycle DNA stain according to the manufacturer’s instructions. Data analysis was completed using FlowJo version.10.8.0 (Treestar).

### Caspase-Glo 3/7 Assay

Caspase-Glo assay was performed as per the manufacturer’s instructions (Promega). Briefly, 5000 cells/well were plated into 96-well plates and treated with a range of compound concentrations. Caspase activity was measured at the noted timepoints based on luminescence read on an Envision 2104 plate reader (PerkinElmer, USA) and normalized to viability.

### Lentiviral Production and DMG Transduction

ShRNA clones targeting *HDAC2* (TRCN0000195198, TRCN0000004823, TRCN0000004819) and *CDKN2A* (TRCN0000281415, TRCN0000039751) were purchased from Sigma-Adrich. For each transduction, 1 million cells were dosed with 5 µL of concentrated lentivirus in DMG culture media supplemented with 1-10 µg/mL of polybrene. After incubating for 36-48 hours, the transduced cells were then harvested for use in downstream experiments.

### Western Blot

Cell pellets were lysed in ice-cold RIPA buffer that was supplemented with protease and phosphatase inhibitors (Life Technologies, cat # 78440) or by boiling in 1x NuPAGE LDS Sample Buffer. Lysates were resolved by SDS-PAGE on NuPAGE 4%-12% Bis-Tris gels (Thermo Fisher Scientific), and wet transferred to PVDF membranes. Primary antibodies are listed in Supplementary Methods. All membranes were imaged on LICOR Odyssey infrared imaging system and analyzed with ImageStudio Odyssey Lite Software (LICOR).

### RNA Sequencing and Analysis

RNA was extracted from DMSO- or Compound 26-treated SU-DIPGXXV cells using RNeasy Plus RNA kit (QIAGEN). Sample purity was verified using an Agilent 2100 Bioanalyzer. Samples were then subjected to library construction with poly-adenylation preparation and sequencing using a NovaSeq 6000 (paired end, 150bp reads). RNA-seq reads were aligned to hg19 using STAR version 2.5.3a. Gene expression values were calculated as Transcription Per Million (TPM) using RSEM version 1.3.0 and the UCSC hg19 reference. DESeq2 was used for differential gene expression analysis. GSEA was done using MSigDB (gsea-msigdb.org).

### AQuA-HiChIP Sequencing and Analysis

Absolute Quantification of Architecture (AQuA)-HiChIP was conducted using the Dovetail MNase-HiChIP kit version 1.3 (Dovetail Genomics, 21007), with specifications outlined in Supplementary Methods.^20^

### CUT&RUN-qPCR Analysis

CUT&RUN was performed using the CUT&RUN kit from EpiCypher with an H3K27ac-specific antibody, followed by qPCR to quantify enrichment at selected genomic regions. Signal was normalized to IgG control and a yeast spike-in control (*ACT1*).

### BBB PAMPA Assay and Mouse PK Studies

The BBB-PAMPA Permeability assay was performed by Creative Bioarray (Shirley, NY, USA) as described in Rabal et al.^21^ Mouse PK testing was performed by ChemPartner using LC/MS-MS.

### H3.3K27M-Glioma Orthotopic Xenograft Models

DIPG-XIIIP* and BT869-luciferase cells were generated as previously described.^22^ All animal studies were performed according to Dana-Farber Cancer Institute Institutional Animal Care and Use Committee (IACUC)-approved protocols, with full details outlined in Supplementary Methods.

### Public Expression and Dependency Data Analysis

Analysis of dependency data were retrieved from the DepMap portal (www.depmap.org) using the 20Q2 dataset. Dependency data for HDAC isoforms were extracted as probability of dependency for all cell lines. Analyses of publicly available expression dataset from Paugh et al. was downloaded from the R2 database (https://hgserver1.amc.nl/cgi-bin/r2/main.cgi#) and proteomic expression was performed using the 20Q1 data release.^23^

### Re-Analysis of Single-Cell RNA-Seq Data

DMG single-cell RNA-seq data were processed as previously published and interrogated for expression of HDAC isoforms in various malignant cell populations using the program Seurat.

### Statistical Analyses

Data from bulk RNA-seq and HiChIP were analyzed as described. Animal experiments were analyzed by the log-rank test for survival. Other data were analyzed with one- or two-sided ANOVA, two-sided t-tests, or one- or two-sided Fisher exact tests as appropriate for multiple or pair wise comparisons. Statistical significance was defined as a *P* < .05 unless otherwise stated. Data were analyzed with GraphPad Prism 9.5.1, and all error bars represent standard deviation unless otherwise noted.

## Results

### HDAC2 is Highly Expressed and a Selective Genetic Dependency in DMG

To determine which HDAC isoforms contribute to DMG growth, we first examined their expression levels in 29 primary DMG samples through bulk RNA expression and in our recently published single-cell RNA sequencing dataset of primary DMG samples.^23^^,^^24^ We found that HDAC2 was expressed at significantly higher levels than all other isoforms except HDAC1 (Figure 1A, B), an isoform essential for neuronal stem cell survival and embryonic viability.^9^ This difference in expression levels in large part comes from the selectively high expression of HDAC2 in cycling, stem-like cancer cells (Figure 1C), which we have previously identified to be the disease-driving population in DMG.^25^ We next sought to determine whether this expression correlated with essentiality using our recently published epigenetic CRISPR/Cas9 screen performed in 5 DMG neurosphere cell lines (Supplementary Table S1).^26^ To assess genetic dependency, we calculated CERES scores that estimate gene dependency levels from CRISPR-Cas9 essentiality screens, with negative CERES scores indicating essentiality. We observed that the Class I HDAC isoforms (HDAC1, 2, and 3) are all important for DMG growth, with HDAC2 and HDAC3 representing bona-fide dependencies (Figure 1D, Supplementary Table S2). Conversely, genetic knockouts of class II/III HDAC isoforms have minimal effect on DMG growth (Figure 1D). Importantly, HDAC3 was found to be a “pan-essential” gene, whose knockout led to loss of fitness or cell death in multiple tissues or cell lineages (Supplementary Figure S1A).^27^ HDAC2, however, emerged as a strongly selective dependency in DMG, as demonstrated by comparing its dependency score to the 317 other available cancer cell lines in the DepMap dataset (Figure 1E-F, Supplementary Figure S1B, Supplementary Table S3).^28^ Additional validation of HDAC2 dependency was performed using shRNA knockdown, which confirmed HDAC2 as a crucial regulator of DMG cell growth (Figure 1G, H, and Supplementary Figure S1C). These results match with prior data showing similar effects of HDAC2 knockdown on DMG viability in additional cell lines (SU-DIPGIV, SU-DIPGVI, SU-DIPGXIII, JHH-DIPG1).^14^ We also validated our findings by exploring HDAC isoform dependencies in the recently published Childhood Cancer Model Atlas (CCMA), which included 39 H3K27M mutant DMG cell lines (Supplementary Figure S1D; Supplementary Table S4).^29^ Together, these results highlight the potential for HDAC1 and HDAC2 having non-overlapping functions in these tumors and establish HDAC2 as a strong and selective dependency in H3K27M-DMG, providing the foundation for subsequent mechanistic and therapeutic investigation.

**Figure 1.**
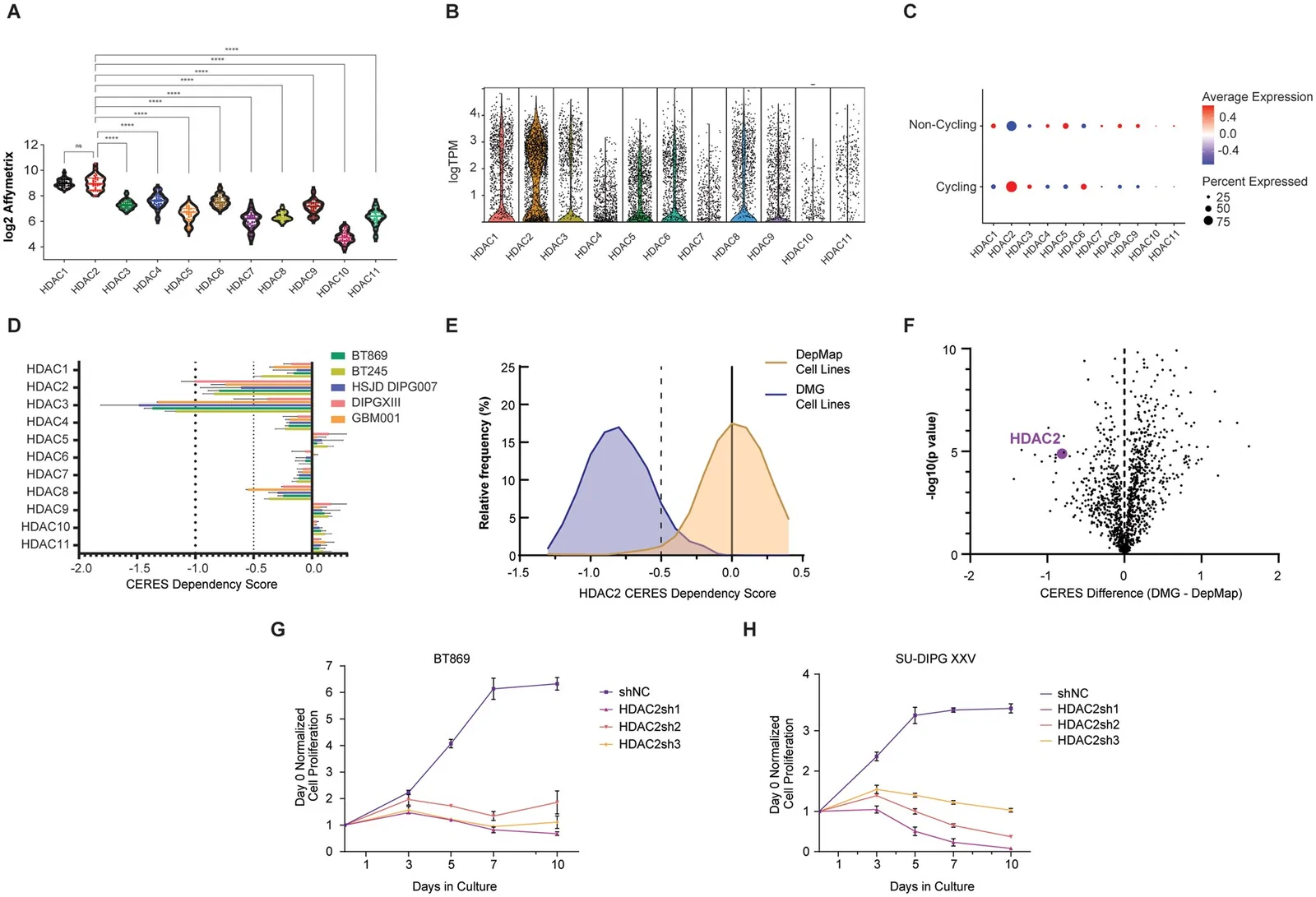
HDAC2 is a selective genetic dependency in H3K27M-DMG. (A) Bulk RNA expression of HDAC isoforms in 29 primary human DMGs measured by affymetrix expression array.^23^ Expression level of HDAC2 vs. other HDAC isoforms was compared using one-way ANOVA with a Dunnett’s multiple comparison test (****P<.0001, ns = not significant). (B) Expression of HDAC isoforms in 6 primary DMG tumors assessed using single-cell RNA sequencing.^24^ (C) Dot plot demonstrating expression scoring for DMG cycling program in single-cell RNA sequencing cohort. (D) CERES dependency scores for HDAC isoforms 1-11 from CRISPR/Cas9 screen. (E) HDAC2 specific histograms of CERES dependency scores for DMG lines compared with 317 cancer cell lines in the DepMap (Broad Institute). (F) CERES difference (geometric mean) for 1248 epigenetically relevant genes from CRISPR/Cas9 screen for DMG cell lines vs. DepMap cancer cell lines. (G) BT869 cell proliferation (assessed using CTG assay) normalized to day 0 with shRNAs targeting HDAC2 or negative controls. Data are expressed as mean ± SD (*n* = 3). (H) SU-DIPGXXV cell proliferation (assessed using CTG assay) normalized to day 0 with shRNAs targeting HDAC2 or negative controls. Data are expressed as mean ± SD (*n* = 3).

### HDAC Class I Isoforms Are Dependencies in DMG That Can be Targeted by Selective Small Molecule Inhibitors

Given the genetic dependence of H3K27M-mutant DMG cells on HDAC2 expression, we hypothesized that selectively inhibiting HDAC2 function in these cells could be an effective therapeutic strategy. To evaluate the utility of such a chemical approach, we employed a small, curated chemical collection containing 8 HDACi with different selectivity profiles: Class Iselective HDAC1/2 inhibitors, Merck 60 and entinostat; HDAC1/2/3 inhibitor, romidepsin; HDAC3-selective inhibitor, RGFP966; non-Class I inhibitors (OJI-1, HDAC8; TMP195, HDAC4/5/7/9; WT161, HDAC6); and pan-HDAC inhibitor (panobinostat).

We first validated compound binding using a well-characterized fluorogenic biochemical assay in which recombinant full-length HDAC proteins catalyze the deacetylation of a synthetic peptide substrate, generating a fluorescent signal proportional to enzyme activity (Figure 2A). The observed potency trends were consistent with previously reported values.^19^ These inhibitors were then tested in a 9-point dose-response assay across 3 H3K27M DMG cell lines, measuring viability after 7 days of treatment. The resulting half maximal inhibitory concentration (IC_50_) values were highly concordant across cell lines and revealed that the pan-HDACi panobinostat, as well as the potent HDAC1/2/3 inhibitor romidepsin were the most potent with IC_50_ values <10 nM (Supplementary Table S5). In contrast, the less biochemically potent HDAC1/2 inhibitors Merck 60 and entinostat displayed moderate cellular potency (IC_50_ ∼200-500 nM), while the HDAC3-selective inhibitor RGFP966 (IC50 = 1-3 μM) and non-Class I inhibitors (OJI-1, TMP195, WT161; IC50 > 5 μM) were far less effective in inhibiting the growth of H3.3 K27M DMG cell lines.^30-32^ Unbiased hierarchical clustering of drug IC_50_ values with their respective isoform selectivity confirmed that only inhibition of class I HDACs robustly inhibited DMG cell growth (Figure 2B). Our findings align with DepMap data showing that HDAC2 gene dependency is correlated with bulk expression and sensitivity to class I HDAC inhibitors like entinostat (Supplementary Figure S2A, B). Similarly, CCMA analysis demonstrated a strong correlation between HDAC2 gene essentiality and entinostat sensitivity across 35 H3K27M mutant DMG cell lines (Supplementary Figure S2C).

**Figure 2.**
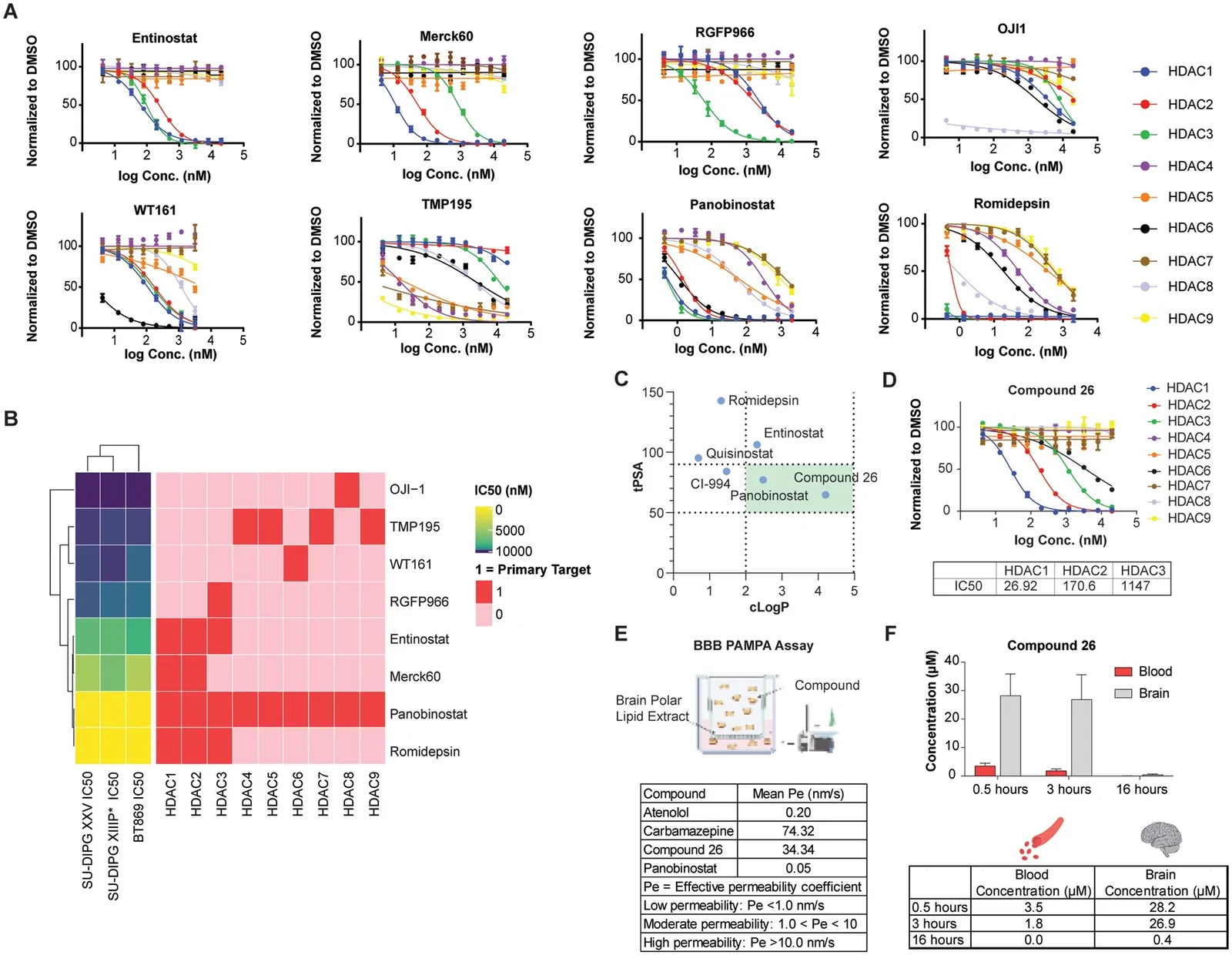
HDAC1/2 isoform catalytic functions are essential in H3K27M-glioma and can be targeted with selective inhibitors. (A) HDAC fluorogenic selectivity profiling for HDAC inhibitors (*n* = 4). Percent HDAC enzyme activity for the indicated HDAC isoforms is reported relative to a DMSO control, as a function of increasing inhibitor concentrations. (B) Seven-day viability screening for selected HDAC inhibitors in three DMG cell lines (*n* = 4). Heatmap depicts unsupervised hierarchical clustering of drug IC_50_ values with isoform selectivity profiles. (C) Plot comparing calculated lipophilicity (clogP) and polar surface area (PSA) of selected HDAC inhibitors, with optimal range for predicted CNS penetrance highlighted in green. (D) HDAC fluorogenic selectivity profiling for Compound 26 (*n* = 4). (E) BBB PAMPA assay schematic (top) and results (bottom) showing permeability coefficients as an *in-vitro* screen for predicted CNS penetrance (*n* = 2). (F) Compound 26 concentration measured in the blood and the brain by mass spectrometry at indicated time-points after 20 mg/kg per os (p.o). administration in non-tumor bearing mice. Data are expressed as mean ± SD (*n* = 3).

After establishing that inhibition of Class I HDACs, particularly HDAC2, most effectively reduces the viability of H3K27M DMG cell lines, we next sought to identify whether these compounds possessed suitable physicochemical properties for central nervous system (CNS) penetration. Because a compound’s ability to cross the BBB is largely determined by its lipophilicity (clogP) and polar surface area (PSA),^33^ we first evaluated these parameters for the class I HDAC inhibitors used in our initial screening. However, none of those compounds fell within the physicochemical space typically associated with BBB permeability (Figure 2C). We therefore extended our profiling to include additional literature-reported Class I-targeting HDAC inhibitors with varying predicted BBB penetration properties.^34-36^ Among these, Compound 26 displayed clogP and PSA values consistent with potential CNS penetration and had previously been shown in non-human primate studies to achieve excellent brain exposure (brain/plasma AUC > 7). Based on these findings, we selected Compound 26 for further characterization.

Using the same fluorogenic biochemical assay as before, we first Compound 26’s selectivity for HDAC1 and HDAC2, observing IC_50_ values of 27 nM and 170 nM, respectively (Figure 2D). The compound also exhibited high predicted BBB penetration in an *in vitro* parallel artificial membrane permeability assay (PAMPA), which measures a compound’s passive permeability across an artificial lipid membrane designed to mimic the BBB (Figure 2E). PK studies in non–tumor-bearing mice further verified strong CNS penetration, with total brain concentrations (∼28 μM) approximately 15-fold higher than plasma levels at 3 hours post-dose, followed by systemic clearance by 16 hours (Figure 2F).

Taken together, our inhibitor profiling and genetic dependency analyses demonstrated that DMG cells selectively rely on class I HDAC activity for survival. By prioritizing compounds with favorable BBB penetration, we identified Compound 26 as a potent, selective HDAC1/2 inhibitor with strong CNS exposure, nominating it for further mechanistic and pharmacological studies as a potential anti-DMG therapeutic.

### Compound 26 Inhibits Growth of DMG Cells through Induction of G1 Cell Cycle Arrest

Having validated the favorable selectivity profile and PK properties of Compound 26, we next evaluated its cellular activity across a broad panel of H3.1/H3.3-mutant and H3K27-wildtype DMG cell lines. Compound 26 showed a significant dose-dependent effect on cell viability, with IC_50_ values ranging from 0.2 to 1 μM, and the H3K27-wildtype DMG lines exhibiting reduced sensitivity at the higher end of this range (Figure 3A). Consistent with prior studies, this attenuated response in H3-wild-type models likely reflects preserved EZH2 function and chromatin stability, as well as the absence of the H3K27M oncohistone, whose expression is selectively reduced by HDAC inhibitors such as panobinostat and entinostat.^37^

**Figure 3.**
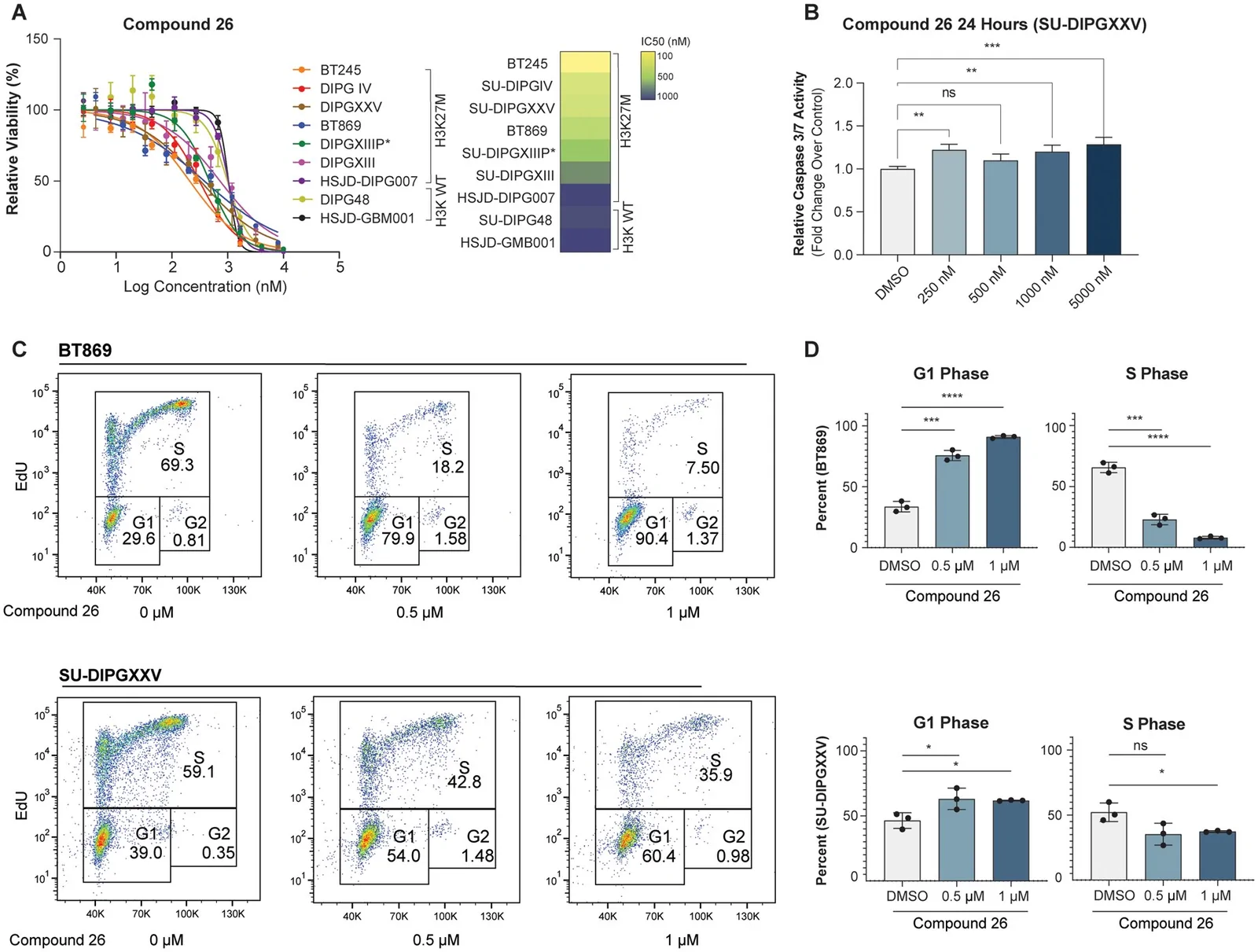
Compound 26 potently inhibits DMG growth and proliferation by inducing G1 cell cycle arrest. (A) Dose-response curves of viability for DMG cell lines treated with Compound 26 (*n* = 4). (B) Relative Caspase 3/7 activity after 24 hours of exposure to Compound 26 at the indicated treatment doses . Statistical comparisons were made using an ordinary one-way ANOVA followed by Dunnett’s multiple comparisons test versus the DMSO vehicle control (*n* = 4). *P <.05 **P <.01, ***P <.001. (C) Representative FACS analysis of proliferation (EdU) and cell cycle composition (FxCycle Violet DNA stain) after three days of Compound 26 treatment. Top = BT869, bottom = SU-DIPGXXV. (D) Quantification of cell cycle composition showing percentage of cells in G1 (left) and S (right) phases. Statistical comparisons were made between Compound 26-treated samples and vehicle controls using two-tailed t-tests (*n* = 3). *P <.05, **P <.01, ***P <.001, ****P <.0001.

To further investigate the mechanism of action, we performed apoptosis and cell cycle analyses in two representative DMG cell lines (BT869, SU-DIPGXXV) following Compound 26 treatment. After three days of exposure, we saw only minimal induction of apoptosis by Caspase 3/7 activity assay or flow cytometry (Figure 3B, Supplementary Figure S3A). However, we observed robust changes in cell cycle distribution, characterized by a significant induction of G1 cell cycle arrest and a corresponding reduction in the proportion of cells in S phase (Figure 3C, D). Consistent with these findings, EdU incorporation assays revealed a marked, dose-dependent decrease in cellular proliferation (Supplementary Figure S3B).

Because HDAC inhibition can disrupt DNA repair and indirectly cause DNA damage by inducing redox stress and double-strand break accumulation, we next assessed whether Compound 26 induced DNA damage in these cells.^38^ Panobinostat, a pan-HDAC inhibitor previously shown to cause extensive DNA double-strand breaks in DMG and other tumor cells, served as a control. Western blot analysis of γH2AX expression revealed that Compound 26 caused substantially less DNA damage than panobinostat (Supplementary Figure S3C), suggesting that selective HDAC1/2 inhibition primarily affects chromatin organization and cell cycle progression rather than directly inducing extensive genotoxic stress. Finally, given that radiotherapy remains the standard-of-care for DMG patients and that HDAC inhibitors can sensitize tumor cells to radiation by impairing DNA repair, we evaluated the combined effects of Compound 26 and radiation in BT869 cells.^39^ We found that co-treatment with Compound 26 at IC_50_-relevant concentrations produced a modest but measurable synergistic effect with 2 Gy irradiation (Supplementary Figure S3D, E).

Collectively, these results demonstrate that selective HDAC1/2 inhibition by Compound 26 suppresses DMG cell growth predominantly through potent G1 cell cycle arrest, with reduced induction of DNA damage, while enhancing the efficacy of radiation therapy.

### Compound 26 Halts Cell Cycle Progression through Upregulation of Key Cell Cycle Regulators

In light of these data, we next sought to determine the mechanism by which HDAC1/2 inhibition regulates cell cycle dynamics in DMG. To achieve this, we performed bulk RNA sequencing after 24 hours of treatment at the drug’s IC_90_ (8 µM) to investigate the transcriptomic changes induced by Compound 26 treatment. Principal component analysis (PCA) revealed marked changes in gene expression signatures, with high correlation between replicates (Figure 4A). Differential expression analysis uncovered 478 genes that were significantly up (181) or down (297) regulated (Figure 4B, Supplementary Figure S4A). Gene set expression analysis identified several notable affected pathways, including expected correlations with canonical HDAC pathway perturbations and multiple components of cell cycle regulation (Figure 4C, Supplementary Figure S4B, Supplementary Table S6-S7). This included upregulation of p53 pathway targets and a corresponding decrease in E2F pathway targets. We confirmed these findings at the protein level, showing a trend towards decreased phospho-Rb and E2F expression and a dramatic increase in the canonical tumor suppressors p16 (*CDKN2A*) and p21 (*CDKN1A*) (Figure 4D). Lastly, we scored the DMG cycling signature derived from our single-cell RNA sequencing atlas in our bulk RNA-seq data and found a significant decrease with Compound 26 treatment (Figure 4E). These results revealed that selective HDAC1/2 inhibition induces the transcriptional modulation of cell cycle pathways leading to growth arrest in DMG.

**Figure 4.**
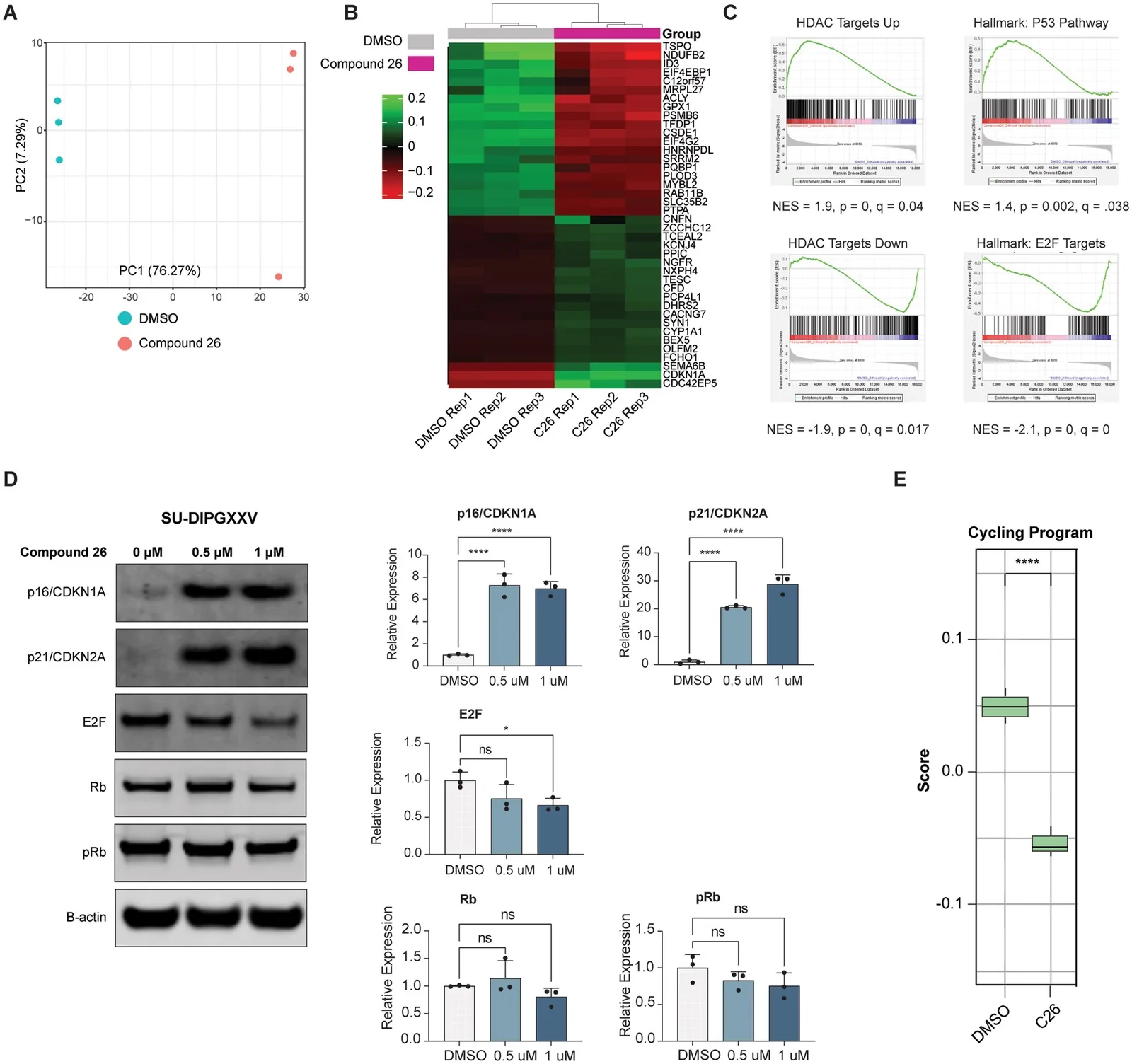
Compound 26 causes profound transcriptional rewiring of DMG cells. (A) Principal component analysis of expression signatures for SU-DIPGXXV cells after 24 hours of treatment with Compound 26 (8 μM, ∼IC_90_) or vehicle control (*n* = 3 per condition). (B) Unsupervised hierarchical clustering of the 100 most differentially regulated genes for each replicate of drug- and control-treated cells. (C) Gene set enrichment analysis (GSEA) identifies several therapeutically relevant pathways affected by Compound 26 treatment. (D) Representative western blot of several key cell cycle regulators after three days of Compound 26 treatment (left). Relative protein quantification (right). Data are expressed as mean ± SD (*n* = 3). *P <.05, ** *P* <.01, *** *P* <.001, **** *P* <.0001. (E) Expression scoring for DMG cycling program (*29*) in Compound 26 and vehicle control samples. **** *P* <.0001 (two-sided *t*-test).

### Interrogation of the Epigenetic Consequences of Compound 26 Treatment Reveals Dramatic Restoration of Physiological Histone Marks

How HDACi-induced hyperacetylation affects chromatin structure to alter gene expression in cancer is poorly understood, and the effects of HDACi are known to be context-specific.^40^^,^^41^ To offer new insights into these questions in the context of Compound 26 treatment, we used AQuA-HiChIP sequencing to map alterations in chromatin 3D structure induced by the compound.^24^ We treated SU-DIPGXXV cells with DMSO or Compound 26 at ∼IC_90_ (8 µM) for 24 hours (same conditions as bulk RNA sequencing), followed by enrichment for H3K27ac and H3K27me3. Upon HDAC inhibition, there was a substantial global increase in H3K27ac as expected, given the drug’s mechanism of action.This was confirmed by western blot analysis and metagene profiling (Figure 5A, Supplementary Figure S5A). Strikingly, we also found a significant global increase in the repressive H3K27me3 mark (Figure 5B, Supplementary Figure S5C), which indicates that Compound 26 overcomes global H3K27me3 hypomethylation, a hallmark of H3K27-altered DMG. Similar effects on H3K27me3 levels were previously observed using pan-HDACi Panobinostat,^14^ suggesting that H3K27me3 restoration induced by pan-HDAC inhibition may primarily be driven by inhibition of HDAC1/2.

**Figure 5.**
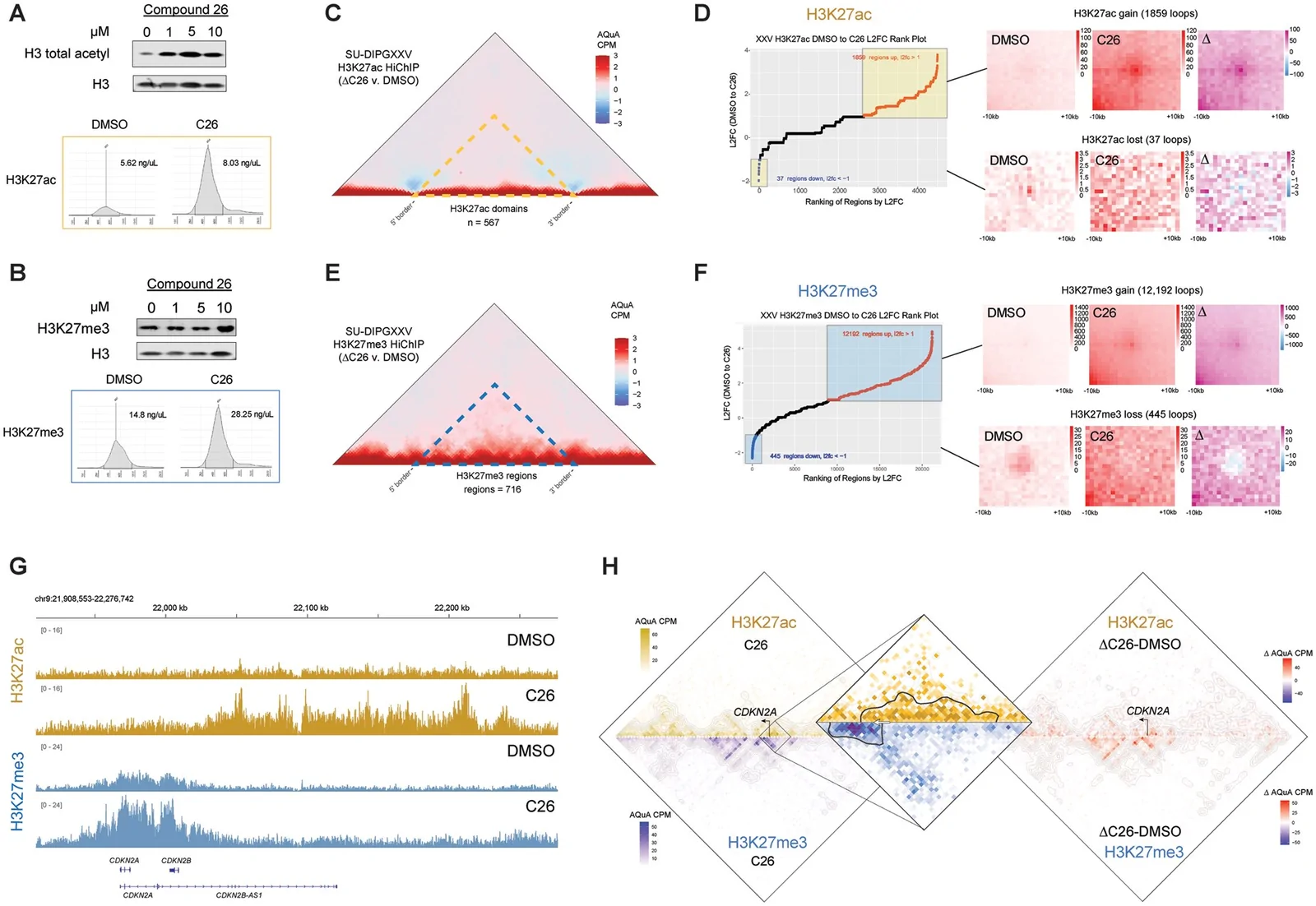
Compound 26 increases H3K27ac and H3K27me3 mediated chromatin contacts. (A) Total acetylated histone H3 western blots (above) and chromatin immunoprecipitated DNA quantities (below) for H3K27ac modified histones, in SU-DIPGXXV cells treated with DMSO or Compound 26. (B) As in (a.), histone K27me3 western blots (above) and chromatin immunoprecipitated DNA quantities for H3K27me3 modified histones. (C) Aggregate topological domain analysis for the change in H3K27ac AQuA-HiChIP signal at H3K27ac domains, comparing DMSO to Compound 26. (D) Loops between H3K27ac peaks in SU-DIPGXXV cells, with the L2FC of loop strength shown ranked (left), and APA (aggregate peak analysis) for DMSO, C26 and the delta of increased and decreased chromatin loops (right). (E) As in (C), aggregate topological domain analysis for the change in H3K27me3 AQuA-HiChIP signal at H3K27me3 domains, comparing DMSO to Compound 26. (F) As in (D), for H3K27me3 AQuA-HiChIP in DMSO- or Compound 26-treated SU-DIPGXXV cells. (G) Genome browser view of H3K27ac and H3K27me3 HiChIP data at the *CDKN2A* gene. (H) Heatmap of AQuA-HiChIP contacts data surrounding *CDKN2A*, in cells treated with Compound 26 (left), and the delta AQuA-HiChIP signal (right).

While the mechanism underlying this phenomenon is still not understood, one model suggests that hyperacetylated H3K27 can “detoxify” K27M-induced inhibition of PCR2.^42^ To probe this mechanism further, we analyzed 3D chromatin dynamics with AQuA contact frequency. Aggregate topological domain analysis between compound-treated and vehicle-treated cells shows a significant gain of H3K27ac diffusely within and outside of topologically associated domain (TAD) borders, which is consistent with known dose-dependent H3K27ac “spreading” effects caused by HDAC inhibition (Figure 5C). We identified 859 drug-specific H3K27ac-loops compared to only 37 for DMSO (Figure 5D). Aggregate topological domain analysis of H3K27me3 after Compound 26 treatment shows a more complex phenomenon, with broad increases in 3D chromatin interactions across TAD boundaries and a net gain of 11,747 H3K27me3 loops (12,192 gained vs. 445 lost; Figure 5E, F).

Given the dramatic upregulation in *CDKN2A* expression after Compound 26 treatment in our bulk RNA-seq data, we hypothesized that this effect was mediated by hyperacetylation at key gene regulatory elements. Indeed, deeper analysis of *CDKN2A* ChIP tracks and contact maps reveals a broad increase in H3K27ac (Figure 5G, H). Notably, this increase is predominantly localized to distal enhancer regions, rather than being confined to the promoter, a finding that was independently validated by CUT&RUN-qPCR in SU-DIPGXXV and BT869 cells (Supplementary Figure S5B). H3K27me3 also increases with Compound 26 treatment, which would seemingly counteract the observed phenotype, but its occupancy was non-overlapping with the increased H3K27ac signal. It has previously been shown that, despite low baseline p16 expression and increased H3K27me3 occupancy at the p16 promoter, pharmacological reduction of methylation with EZH2 inhibition fails to restore p16 expression.^43^ These findings suggest that additional epigenetic mechanisms contribute to H3K27M-mediated p16 repression. Notably, prior studies have demonstrated that genetic knock-in of p16 expression is sufficient to induce growth arrest in DMG cells, consistent with our observation that selective HDAC1/2 inhibition induces a similar effect through hyperacetylation at the p16 locus.^44^ To directly address the contribution of p16 restoration to the efficacy of Compound 26 in DMG, we performed shRNA-mediated knockdown of p16 in BT869 cells, which partially attenuated Compound 26 potency relative to negative controls (Supplementary Figure S5D, E, Supplementary Table S8).

We further confirmed widespread increases in H3K27ac after Compound 26 treatment across additional cell cycle regulator gene bodies, including *CDKN1A* and *CDKN2D*, both of which were upregulated in our RNA-seq dataset (Supplementary Figure S5F). Collectively, these results suggest that Compound 26 induces a global increase in both H3K27ac and H3K27me3 marks. While elevated H3K27ac levels are consistent with the expected on-target effects of HDAC inhibition, the concurrent increase in H3K27me3, though previously reported with pan-HDAC inhibitors in H3K27M-mutant DMG, remains mechanistically unresolved.^14^^,^^45^ Previous studies have proposed that this phenomenon may reflect partial restoration of EZH2 activity and redistribution of H3K27 methylation across the genome. Consistent with this model, we observed significant alterations in 3D chromatin organization, particularly at topologically associating domain (TAD) boundaries, suggesting that HDAC inhibition-induced H3K27ac “spreading” may reorganize chromatin architecture in a manner that promotes renewed EZH2 recruitment and localized H3K27me3 deposition.

### Compound 26 Demonstrates Efficacy in H3K27-Altered DMG Intracranial Orthotopic Models

We next wanted to evaluate the PD markers and therapeutic potential of Compound 26 in an aggressive patient-derived xenograft (PDX) model (SU-DIPG XIIIP*, a fast-growing subclone of the SU-DIPG XIII cell line) engineered to express firefly luciferase. Tumor cells were orthotopically implanted into the pons of immunocompromised NSG mice, and tumor establishment and progression were monitored by bioluminescence (BLI) imaging. Mice were randomized to receive Compound 26 or vehicle control, with Compound 26 administered orally at 20 mg/kg per dose, twice daily on a 5 days-on/2-days-off schedule. Following confirmation of good tolerability and stable body weight, the dose was escalated to 40 mg/kg per dose, administered twice daily beginning in week 2 of treatment (Supplementary Figure S6A).

We observed a reduction in absolute BLI after 2 weeks of treatment (Supplementary Figure S6B; *P *= .0404), along with a consistent trend toward reduced relative BLI at each assessed time point (Figure 6A). Treatment with Compound 26 significantly extended survival compared to vehicle control (*P* < .001 by log-rank analysis; Figure 6B). PD analyses were performed by sacrificing 1 mouse from each cohort after 5 days of treatment. Histological examination revealed highly proliferative tumors in both treatment arms as early as 14 days post-implantation, underscoring the aggressive nature of this model (Figure 6C). Consistent with the BLI findings, tumor volume was smaller in the Compound 26-treated mouse relative to vehicle control, supporting an inhibitory effect on tumor growth and proliferation. In agreement with our *in vitro* data, immunostaining for acetylated-H3 demonstrated a marked increase in the Compound 26-treated tumor (Figure 6D). To validate these findings, we assessed H3 acetylation in a larger cohort of mice from each treatment arm at neurologic endpoints and confirmed significantly higher H3-acetyl levels in Compound 26-treated tumors compared with controls (*P* < .001, Figure 6E).

**Figure 6.**
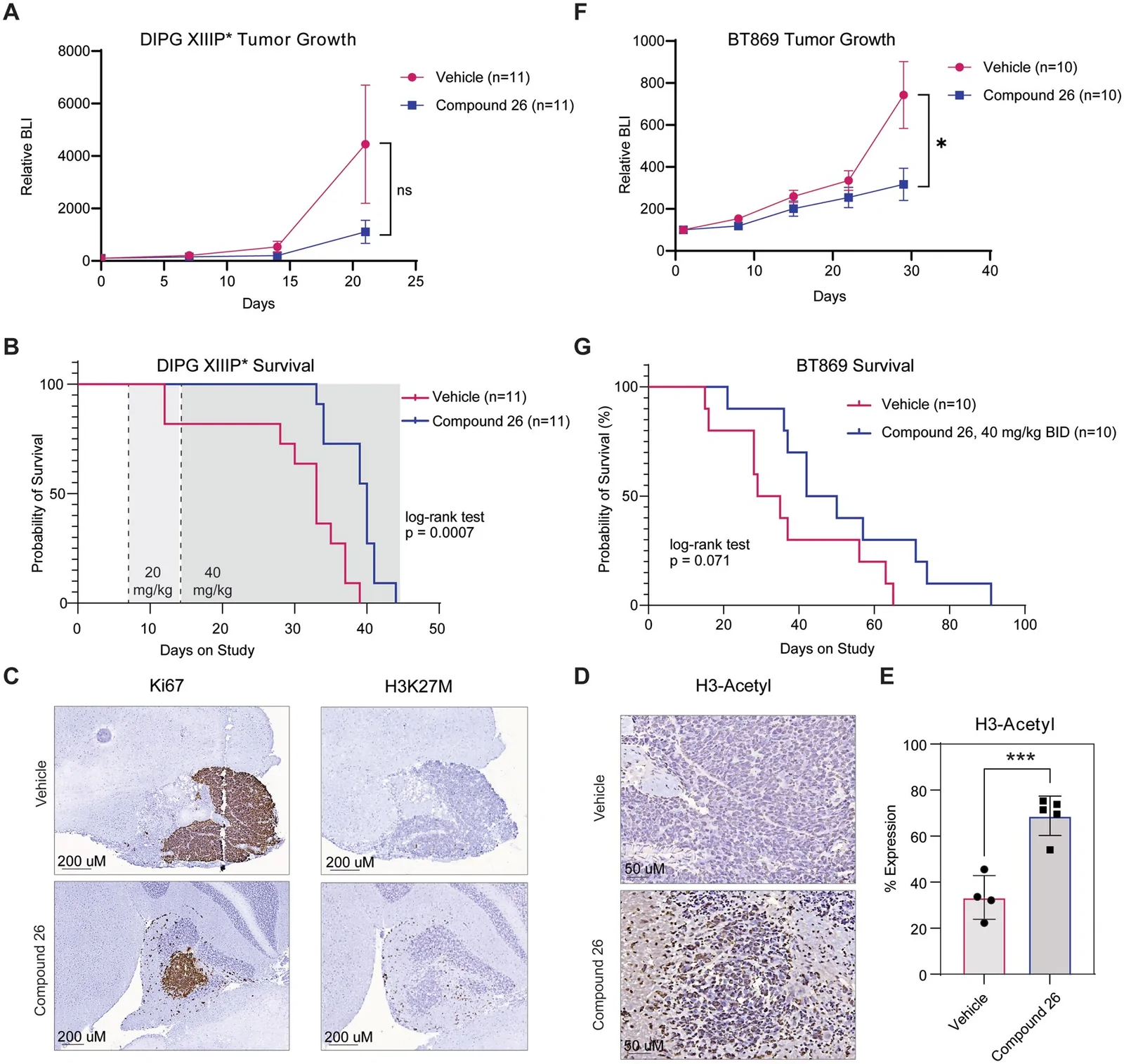
Compound 26 crosses the blood-brain barrier and exerts anti-tumor efficacy in DMG xenograft models. (A) Quantification of *in-vivo* bioluminescence measurements in DIPGXIIIP* tumor-bearing mice treated with Compound 26 or vehicle control through 21 days of treatment (*n* = 11, *P* = .11 at 21 days). (B) Kaplan Meier survival curves of mice bearing DIPG XIIIP* tumors treated with Compound 26 or vehicle (*n* = 11 per group). (C) Immunohistochemistry of DIPGXIIIP* PDX model showing Ki67 and H3K27M co-staining to define tumor location and size. (D) Immunohistochemistry of DIPGXIIIP* PDX model showing increased histone H3 acetylation in Compound 26-treated mice compared with vehicle control. (E) Quantification of H3-Acetyl immunohistochemical signals in Compound 26- (*n* = 5) and vehicle- treated (*n* = 4) mice, shown as mean ± SD; **P <.001 (two-sided *t*-test). (F) Quantification of *in-vivo* bioluminescence measurements in BT869 tumor-bearing mice treated with Compound 26 or vehicle control through 29 days of treatment (*n* = 10, *P* =.019 at 29 days). (G) Kaplan Meier survival curves of mice bearing BT869 tumors treated with Compound 26 or vehicle (*n* = 10 per group).

We next evaluated Compound 26 in a second H3K27M PDX model, BT869, which exhibits a longer latency period of approximately 3 months. Following the detection of established tumors by BLI, mice were randomized to receive Compound 26 (40 mg/kg per dose, administered orally twice daily for 5-days-on/2-days-off schedule) or vehicle control. Tumor growth, as assessed by BLI, was significantly reduced in the Compound 26-treated group, accompanied by a non-significant trend toward prolonged survival (Figure 6F, G). Collectively, these findings support the therapeutic potential of targeting DMG with HDAC Class-I selective, CNS-penetrant HDAC inhibitors.

## Discussion

DMGs are driven by the H3K27M oncohistone and are therefore intrinsically poised for sensitivity to epigenetic therapies. Indeed, inhibitors targeting diverse epigenetic regulators, including HDACs, lysine-specific demethylase 1 (LSD1), EZH2, bromodomain and extra-terminal domain (BET), and DNA methyltransferase (DNMT), have demonstrated promise in this disease.^44-47^ Given the impressive preclinical activity of HDAC inhibitors in DMG, a systematic examination of the therapeutic relevance of individual HDAC isoforms in DMG is long overdue. Here, we demonstrate that HDAC expression in H3K27M DMG is not uniform and identify HDAC2 as a selective and therapeutically relevant dependency in this disease. We find that HDAC2 is highly expressed in DMG cells, enriched within tumor-driving cycling cell populations, and is essential for cell survival. Consistent with this dependency, pharmacologic profiling using a panel of HDAC inhibitors revealed that compounds targeting Class I HDACs, particularly those with HDAC1/2 selectivity, exhibit enhanced potency in DMG cell lines. Although the dual activity of currently available inhibitors precludes definitive attribution of these effects exclusively to HDAC2, the convergence of our genetic and pharmacologic data strongly implicates HDAC2 as a selective vulnerability in DMG. Importantly, this sensitivity appears specific to H3K27M-mutant tumors, as H3-wild-type models display reduced responsiveness to HDAC inhibition.

While HDAC1 and HDAC2 are frequently described as functionally redundant due to their high structural homology and shared incorporation into transcriptional repressor complexes such as NuRD, Sin3, and CoREST, accumulating evidence suggests that they also perform distinct, non-overlapping roles across developmental stages and disease contexts. Our observation that HDAC2, but not HDAC1, is critical for DMG cell survival likely reflects a unique requirement for specific HDAC2-mediated processes that maintain tumor proliferation and viability. This oncogenic dependency mirrors normal brain development, where HDAC2, but not HDAC1, is required for neural stem cell survival and proliferation, highlighting a potential convergence of developmental and tumorigenic programs in DMG.

Mechanistically, we show that selective HDAC1/2 inhibition suppresses DMG proliferation by inducing G1 cell cycle arrest. Transcriptomic analyses reveal marked downregulation of cell cycle-related pathways and disruption of DMG-specific cycling programs. Using AQuA-HiChIP sequencing, we demonstrate that this effect is mediated by concurrent increases in both H3K27ac and H3K27me3 marks, which induce large-scale chromatin reorganization. While elevated H3K27ac levels are consistent with the expected biochemical consequences of HDAC inhibition, the accompanying increase in H3K27me3 levels likely reflects partial restoration of EZH2 activity and subsequent redistribution of H3K27 methylation across the genome. Moreover, our data indicate that the previously described “detoxification” of H3K27M-induced PRC2 inhibition—observed with pan-HDACi—is primarily driven by HDAC1/2 inhibition.^18^^,^^46^ Extending these findings, our 3D chromatin analyses reveal that HDAC1/2 inhibition promotes H3K27ac spreading across topologically associating domain (TAD) boundaries and the formation of more defined chromatin loops. We propose that these acetylation-driven structural changes facilitate renewed recruitment of EZH2 and localized H3K27me3 deposition.

From a translational perspective, poor CNS penetration has been a major obstacle limiting the clinical success of earlier HDAC inhibitors, such as panobinostat. Both preclinical and clinical studies (e.g. PBTC-047) have demonstrated that systemically delivered panobinostat achieves subtherapeutic concentrations in the brain and exhibits limited antitumor efficacy.^48^ In contrast, repeated convection-enhanced delivery of aqueous panobinostat (MTX110) directly into the brains of DMG patients improved intratumoral exposure and extended median survival by 26 months, underscoring the importance of achieving sustained on-target CNS drug delivery.^49^ Guided by these insights, we focused on identifying HDAC1/2-selective compounds with favorable BBB permeability and PK properties. Through this effort, we identify Compound 26, which combines potent HDAC1/2 selectivity with high CNS penetration and favorable tolerability under a twice-daily 5 days per week dosing regimen. These properties translated into promising *in vivo* efficacy in two aggressive orthotopic PDX models of DMG.

Nevertheless, the modest survival benefit observed in our preclinical models suggests that the current potency of Compound 26 may be insufficient to sustain prolonged suppression of HDAC2 function at therapeutically effective levels within the brain. This limitation reflects a broader challenge in the field: the high structural similarity between HDAC1 and HDAC2 complicates efforts to achieve stronger isoform selectivity while enhancing CNS exposure and tolerability. Despite this challenge, further optimization of Compound 26 remains feasible. Structural refinements that enhance and prolong target engagement, rather than simply increasing peak inhibition, may enhance functional potency in the CNS. In parallel, chemical modifications aimed at improving metabolic stability and brain retention could further enhance effective CNS exposure without exacerbating systemic toxicity. While the development of truly HDAC2-selective, CNS-penetrant inhibitors remains an important goal, HDAC1/2-selective compounds currently represent the most tractable path toward clinical translation.

Given the modest survival benefit we observed with monotherapy, combination strategies will likely be required to achieve durable therapeutic responses. Several targets—AXL, BRD4, CDK7, KDM1A/LSD1, DNMT, PI3K, FACT, PDGRFA, and the proteasome—have shown potential synergy with HDAC inhibition in DMG and warrant further investigation.^50^ Consistent with this rationale, we demonstrate that Compound 26 modestly synergizes with radiation *in vitro*, supporting radiotherapy as another promising combinatorial approach. As Compound 26 has not yet entered clinical testing, additional optimization and development will be required, including formulation, pharmacological characterization, and comprehensive safety studies.

In summary, we identify HDAC2 as a critical and therapeutically tractable dependency in DMG. Future efforts will require further chemical optimization, rigorous pharmacologic evaluation, and preclinical safety studies to advance HDAC2-selective compounds toward clinical translation.

## Supplementary Material

noag142_Supplementary_Data

## Data Availability

All data are provided in the main manuscript, the supplementary materials or through publicly accessible domains. AQuA-HiChIP data from this study are available in the Gene Expression Omnibus (GEO) database and can be accessed with accession number **GSE273609**.
