# Supplementary material for: Selective histone deacetylase inhibition as a therapeutically relevant strategy in H3K27M-glioma
Source: Neuro Oncol. 2026 Jun 25;28(10):2485–99. doi: 10.1093/neuonc/noag142 (PMC13635581; doi:10.1093/neuonc/noag142)
Supplement: Supplementary file 1 [file noag142_supplementary_data.zip › Supplementary_Data.zip!/Supplementary_Data/Supplementary Figures with Legends.docx]

 **Supplementary Figure 1: HDAC2 is a selective dependency in DMG cell lines, with HDAC3 representing a pan-essential gene**

(A) CERES dependency scores of HDAC isoforms 1-11 from 317 adult and pediatric cancer cell lines in the DepMap (Broad Institute).
(B) HDAC isoform essentiality and selectivity across DepMap cell lines (shinyDepMap).
(C) Related to Figure 1 G/H: Efficacy of HDAC2 shRNA knockdown in BT869 and SU-DIPGXXV assessed using western blot.
(D) HDAC isoform CRISPR z scores for 39 H3K27M mutant DMG cell lines from the Childhood Cancer Model Atlas.^29^

**Supplementary Figure 2: HDAC2 expression and sensitivity to class I inhibitors is associated with genetic dependency**

(A) HDAC2 gene effect vs. RNA expression in adult and pediatric DepMap cell lines.
(B) HDAC2 gene effect vs. Entinostat AUC in adult and pediatric DepMap cell lines (data available from DepMap and PRISM screens, respectively).
(C) HDAC isoform CRISPR essentiality and Entinostat drug screen z-scores for 35 H3K27M mutant DMG cell lines from the Childhood Cancer Model Atlas.

**Supplementary Figure 3: Compound 26 induces mild pro-apoptotic effect, but dramatically stalls proliferation**

(A) Representative FACS analysis of apoptosis by AnnexinV/7AAD in BT869 cells after three days of Compound 26 treatment at the indicated doses.
(B) Histogram of EdU incorporation by flow cytometry after three days of Compound 26 treatment at the indicated doses. Left = BT869, right = SU-DIPGXXV.
(C) γ-H2AX staining of DIPG XXV after Compound 26, panobinostat treatment at indicated doses for 48 hours.
(D) Cell viability of BT869 cells after six days of treatment with Compound 26 (0, 200 nM, or 400 nM) in combination with radiation (0, 2, or 4 Gy) in a dose–response matrix format (*n* = 3).
(E) Data from Supp Fig. 3D plotted as HSA synergy map using SynergyFinder3.0.

**Supplementary Figure 4: Compound 26 induces strong transcriptomic effects on cell cycle regulation**

(A) Volcano plot demonstrating significantly up- and down-regulated genes with Compound 26 treatment.
(B) Dot plot summarizing top 10 significant enrichments (*P* ≤ 0.05) for the Gene Ontology biologic processes annotations.

**Supplementary Figure 5: Compound 26 induces global increases in H3K27ac and H3K27me3**

(A) Metagene profile plot of H3K27ac HiChIP data along the body of genes upregulated by Compound 26, in SU-DIPGXXV cells treat with DMSO or Compound 26 for 24 hours.
(B) Normalized changes in H3K27ac occupancy at the p16 promoter, gene body and distal enhancer regions following CUT&RUN-qPCR in SU-DIPGXXV (*n* = 2) and BT869 (*n* = 3) cells treated with Compound 26 (8 µM) or DMSO control for 24 hours. Data are expressed as the mean ± SE for the indicated number of biological replicates, and statistical significance is determined using 2-way ANOVA. **P* < 0.0322, ***P* < 0.0021.
(C) Metagene profile plot of H3K27me3 HiChIP data along the body of all genes, in SU-DIPGXXV cells treat with DMSO or Compound 26 for 24 hours.
(D) Normalized p16 mRNA expression fold change with shRNA knockdown of p16 using two lentiviral vectors in BT869.
(E) DMSO Normalized cell proliferation of BT869 cells after lentiviral knockdown and subsequent treatment with Compound 26 at indicated doses for 3 days (*n* = 3). Normalized cell proliferation of p16 shRNA knockdown BT869 cells compared to negative control using 2-way ANOVA.
(F) Genome browser view of H3K27ac HiChIP and RNA-seq data at the CDKN2D and CDKN1A genes.

**Supplementary Figure 6: Compound 26 is well tolerated in mice and slows *in-vivo* DMG growth**

(A) Body weight measurements in DIPG XIIIP* tumor-bearing mice treated with either vehicle control or Compound 26. Compound 26 dosing started at 20 mg/kg/dose oral twice daily for 5 days­­­­ on/2 days off. After one week dosing was increased to 40 mg/kg/dose oral twice daily and continued for three weeks. Data are expressed as mean ± SD (*n* = 11).
(B) *In vivo* bioluminescence imaging of mice xenografted with DIPG XIIIP*-luciferase cells immediately before or 14 days after initiation of treatment with Compound 26 or vehicle control. Data are expressed as mean ± SD. Statistical significance was assessed using two-tailed unpaired t-tests. **P* = 0.0404
