# Supplementary material for: Selective histone deacetylase inhibition as a therapeutically relevant strategy in H3K27M-glioma
Source: Neuro Oncol. 2026 Jun 25;28(10):2485–99. doi: 10.1093/neuonc/noag142 (PMC13635581; doi:10.1093/neuonc/noag142)
Supplement: Supplementary file 1 [file noag142_supplementary_data.zip › Supplementary_Data.zip!/Supplementary_Data/Supplementary Methods.docx]

**Supplemental Materials and Methods**

**Cell Culture**

H3K27M-glioma cells were grown as neurospheres in tumor stem media (TSM) base supplemented with B27 minus vitamin A (Thermo Fisher Scientific), human growth factors (EGF, FGF, PDGF-AA, PDGF-BB [Shenandoah Biotechnology]) and heparin (Stemcell Technologies) in ultra-low attachment flasks. All neurosphere models were authenticated by high resolution short tandem repeat (STR) profiling and routinely checked for mycoplasma contamination (Lonza).

**Drug Testing and Cell Viability Assays**

Merck 60, “Compound 26”, WT161, and OJI-I were synthesized in the Qi lab. Compound structure and purity were confirmed by nuclear magnetic resonance and LC/MS. Panobinostat, Entinostat and TMP195 were purchased from SelleckChem. RGFP966 was purchased from Thermo Fisher Scientific, and Romidepsin was purchased from SigmaAldrich. For radiation experiments, BT869 cells were pre-treated with Compound 26 at indicated concentrations in 96-well plates and then irradiated using a Small Animal Radiation Research Platform (SARRP; Xstrahl, Suwanee, GA, USA) equipped with a drawer-style irradiation chamber. The plates were placed inside the chamber and exposed to a single dose of 2 Gy or 4 Gy at room temperature. System output and dose delivery were verified according to institutional dosimetry procedures prior to irradiation. Non-irradiated cells served as 0 Gy controls. Synergy plots generated using SynergyFinder3.0 ( [https://synergyfinder.fimm.fi](https://synergyfinder.fimm.fi/)).

**Lentiviral Production and DMG Transduction**

For lentiviral production, 90% confluent HEK 293T cells in 100 mm plates were transfected with pMD2.G (3 µg), psPAXΔ2 (9 µg) and shRNA plasmid (9 µg) using polyethyleneimine as the transfection reagent. The culture media was refreshed 12-16 hours. The lentivirus-containing supernatant was then harvested 48- and 72-hours post-transfection, pooled, filtered through a 0.45 µm filter and concentrated by centrifugation at 8000 x g overnight at 4 ℃. The lentiviral pellets were then each resuspended in 120 µL of TSM base media per initial 100 mm plate and then stored at -80℃ until needed for transduction.

Verification of knockdown was performed by western blot and/or Reverse Transcription-Quantitative Polymerase Chain Reaction (RT-qPCR) Assay. For RT-qPCR: Total RNA was extracted using the RNeasy Plus Kit (Qiagen) according to the manufacturer’s protocol. RNA concentration and purity were determined using a NanoDrop spectrophotometer (Thermo Fisher Scientific). Subsequently, 700 ng of total RNA was reverse transcribed into cDNA using the Quantitect Reverse Transcription Kit (Qiagen) following the manufacturer’s instructions. Quantitative PCR was performed in triplicates on a ViiA 7 Real-Time PCR System (Thermo Fisher Scientific) using SYBR Green Chemistry (PowerUp SYBR Green Master Mix for qPCR, Applied Biosystems). Gene expression was quantified using the ΔΔCt method, normalized to the expression of *ACTB*, and presented relative to the non-targeting control shRNA. The primer sequences used are listed in Supplementary Table 8.

To evaluate the effect of HDAC2 knockdown on DMG cell proliferation, cells were harvested 36–48 hours after shRNA transduction, and 5,000 cells were seeded per well in quadruplicate in 96-well plates containing fresh media. Cell proliferation was assessed over 7 days using CellTiter-Glo® reagent (Promega) according to the manufacturer’s instructions. Cell viability was measured on the day of plating (day 0) and on days 3, 5, and 7. Luminescence was recorded using a CLARIOstar plate reader, and values were normalized to day 0.

To investigate the impact of Compound 26 treatment on DMG cell proliferation following *CDKN2A* knockdown, 5,000 transduced cells were plated per well in triplicate in 96-well plates. After overnight incubation, cells were treated with Compound 26 at final concentrations of 0 μM (DMSO control), 1 μM, 5 μM, or 10 μM. Cells were incubated for 6 days, and viability was then measured using CellTiter-Glo® as described above. Values were normalized to the DMSO control.

**Western Blot**

Primary antibodies used include: HDAC2 (Cell Signaling Technology, Cat #5113S0), Rb (Cell Signaling Technology, Cat #9313), phospho-Rb (Cell Signaling Technology, Cat #8516), E2F (Cell Signaling Technology, Cat #3742), p16 (Cell Signaling Technology, Cat #80772), p21 (Cell Signaling Technology, Cat #2947), H3-acetyl (K9+K14+K18+K23+K27; Abcam, Cat #47915), H3K27ac (Abcam, Cat #ab4729), H3K27me3 (Cell Signaling Technology, Cat #9733S), H3-total (Cell Signaling Technology, Cat #9715), yH2AX (Cell Signaling Technology, Cat # 80312S), GAPDH (Cell Signaling Technology, Cat #5174S)and B-actin (Cell Signaling Technology, Cat # 3700). LICOR goat anti-rabbit IRDye® 800 (Licor, Cat #926-32211) or goat anti-mouse IRDye®680 (Licor, Cat #926-68070) antibodies were diluted at 1:10:000 in TBS-T or 1:5000 in PBST and incubated at room temperature for 1 hour.

**AQuA-HiChIP Sequencing and Analysis**

Absolute Quantification of Architecture (AQuA)-HiChIP was conducted using the Dovetail MNase-HiChIP kit version 1.3 (Dovetail Genomics, 21007) with the following specifications.^24^ For each experiment, 10x10^6^ SU-DIPGXXV cells were treated with Compound 26 (8 µM) for 24 hours, washed, and flash frozen. Cells were then thawed and fixed with DSG and 1% formaldehyde, then digested with MNase for 15 minutes at 22˚C and stopped with EGTA. Cells were then lysed with RIPA buffer, and chromatin was evaluated for digestion efficiency, achieving 50-70% mononucleosomes. Immunoprecipitation was conducted with anti-H3K27ac (Millipore, MABE647) and anti-H3K27me3 (Cell Signaling Technology, 9733). Spike-in for AQuA-normalization was performed by adding chromatin extracted from C2C12 cells at a defined and conserved ratio of 1:10 mouse chromatin to human chromatin. After immunoprecipitation, samples were washed twice with RIPA buffer, twice with high salt (400mM NaCl) wash buffer, twice with LiCl (250mM) wash buffer, and twice with Dovetail 1x wash buffer (150 mM NaCl). Bead-bound chromatin was then end polished, followed by ligation of biotin-containing bridge DNA, crosslink reversal and proteinase K digestion, and DNA purification with SPRI select beads. Libraries were prepared by ligating Illumina adaptors, followed by binding to streptavidin beads, washing, and performing on-bead barcoded index PCR.

Each AQuA-HiChIP sample was sequenced to a depth of 300 million or more paired-end reads with an Illumina NovaSeq 6000. Paired-end reads were aligned to human genome build hg38 and mouse genome build mm10 in parallel, using Bowtie2 as part of the HiC-Pro pipeline. The significance of peaks was tested using MACS3 (version 3.0.0a6, <https://github.com/taoliu/MACS>) with the -B option, a regular TF-binding mode. Peak artifacts were removed (reference locations “black-listed” by the ENCODE consortium <https://sites.google.com/site/anshulkundaje/projects/blacklists>, ENCFF356LFX). Mapped reads were then filtered for junction validity and read pairs with contact range of 1000 bp or less were removed to filter out spurious MNase ligation products. Duplicate read-pairs of HiChIP pull-downs were not removed as they tended to pile up at ChIP-seq peaks. Final read pairs (all valid pairs) were converted to Juicebox compatible .hic files for visual inspection using the hicpro2juicebox script provided within HiC-Pro. Downstream analyses requiring sub-matrix extraction from .hic files were performed using Juicer and strawr.

**CUT&RUN-qPCR Analysis**

Cells were harvested using Accutase (STEMCELL Technologies), washed with TSM base media, and counted. For each CUT&RUN reaction, 500,000 cells (with ~10% excess) were pelleted and resuspended in fresh TSM buffer prior to fixation. To preserve chromatin–protein interactions, cells were fixed with 1% formaldehyde for 1 min at room temperature, and crosslinking was quenched with 125 mM glycine for 5 min. Following fixation, cells were washed twice with PBS and subjected to nuclei isolation by incubation on ice for 10 min in nuclei extraction buffer (20 mM HEPES pH 7.5, 10 mM KCl, 0.1% Triton X-100, 0.01% BSA, 0.01% Tween-20, 20% glycerol, 1× protease inhibitor cocktail, and 0.5 mM spermidine). Nuclei were then pelleted by centrifugation and resuspended in fresh extraction buffer for downstream processing.

CUT&RUN was performed using the CUT&RUN kit from EpiCypher (v6) according to the manufacturer’s instructions. Briefly, nuclei were immobilized on concanavalin A–coated magnetic beads and incubated with an H3K27ac antibody (EpiCypher, #13-0059) or IgG control antibody (EpiCypher, # 13-0042), followed by targeted chromatin cleavage using protein AG-MNase. The released DNA fragments subsequently underwent reverse-crosslinking and were then purified using the CUTANA DNA Purification beads (EpiCypher).

For qPCR analysis, purified CUT&RUN DNA was quantified on a Qubit 2.0 fluorometer using the Qubit High Sensitivity dsDNA assay kit (Thermo Fisher Scientific). DNA was then used for quantitative PCR using PowerUp SYBR Green Master Mix (Applied Biosystems) on a ViiA 7 Real-Time PCR System (Thermo Fisher Scientific), following standard cycling conditions recommended by the manufacturer. Primer sequences for genomic loci analyzed are provided in Supplementary Table 9. Enrichment at target regions was calculated using the ΔΔCt method relative to IgG controls. Signal normalization across experiments was performed using the *ACT1* spike-in control (Active Motif, #71033).

**Blood-Brain Barrier PAMPA Assay and Mouse Pharmacokinetic Studies**

The BBB-PAMPA Permeability assay was performed by Creative Bioarray (Shirley, NY, USA) as described in Rabal et al.^25^ Compounds were tested at a concentration of 10 μM with an incubation time of 4 hours. Atenolol and Carbamazepine were utilized as negative and positive controls, respectively.

For mouse pharmacokinetic testing, nine CD1 mice (Charles River Laboratories) were administered Compound 26 PO at a dose of 20 mg/kg, resuspended in a 2 mg/mL solution of 5% DMSO:95% methylcellulose. Blood concentration of Compound 26 was measured at the three indicated time points by LC/MS-MS. Brain concentration of Compound 26 was measured by sacrificing mice at the indicated time points followed by snap freezing of whole brain, homogenization in 3 volumes of PBS and analysis by LC/MS-MS. Pharmacokinetic parameters were calculated with WinNonlin V 6.2 software (Pharsight Corporation) using a noncompartmental model.

**H3.3K27M-glioma Orthotopic Xenograft Models**

DIPG-XIIIP*-luciferase or BT869-luciferase neurosphere cells were injected stereotactically into the right pons of 6 weeks-old female NSG mice treated with buprenorphine 0.05mg/kg and anesthetized with isoflurane 2–3%. For DIPG-XIIIP* model, given short latency treatment was started at one-week post-injection Compound 26 (20 mg/kg twice daily 5 days/week x 4 weeks) or vehicle control. Dosing was increased to 40 mg/kg/dose twice daily starting week 2 of treatment after mice were found to tolerate treatment well with stable weight. For BT869 model, once tumor engraftment was confirmed by bioluminescence imaging, mice were randomized based on normalized bioluminescence signal to a treatment arm (40 mg/kg/dose twice daily vs. vehicle control). In both arms, mice were euthanized, and mouse brains were collected and frozen in OCT. Blocks were sectioned into four-micron thick sections and stained for hematoxylin and eosin (H&E), Ki67 (Abcam, ab16667), H3K27M (Abcam, ab190631), and H3-acetyl (Abcam, ab47915) by iHisto histopathology support. IHC quantification was performed using QuPath v0.3.0.
